# Treatment of Heart Failure with Mid-Range Ejection Fraction: What Is the Evidence

**DOI:** 10.3390/jcm10020203

**Published:** 2021-01-08

**Authors:** Eleni-Evangelia Koufou, Angelos Arfaras-Melainis, Sahil Rawal, Andreas P. Kalogeropoulos

**Affiliations:** 1 Division of Cardiology, University of Patras, 26504 Rio, Greece; elenikoufou17@gmail.com; 2 Department of Medicine, Jacobi Medical Center, Albert Einstein College of Medicine, New York, NY 10461, USA; angelosarfaras@gmail.com; 3 Department of Medicine, Stony Brook Renaissance School of Medicine, Stony Brook, NY 11794, USA; sahil.rawal@stonybrookmedicine.edu; 4 Division of Cardiology, Department of Medicine, Stony Brook Renaissance School of Medicine, Stony Brook, NY 11794, USA

**Keywords:** heart failure, heart failure with mid-range ejection fraction, therapy, left ventricular systolic function, beta blockers, angiotensin-converting enzyme inhibitors, angiotensin receptor blockers, mineralocorticoid receptor antagonists, survival, outcomes

## Abstract

In this review, we briefly outline our current knowledge on the epidemiology, outcomes, and pathophysiology of heart failure (HF) with mid-range ejection fraction (HFmrEF), and discuss in more depth the evidence on current treatment options for this group of patients. In most studies, the clinical background of patients with HFmrEF is intermediate between that of patients with HF and reduced ejection fraction (HFrEF) and patients with HF and preserved ejection fraction (HFpEF) in terms of demographics and comorbid conditions. However, the current evidence, stemming from observational studies and post hoc analyses of randomized controlled trials, suggests that patients with HFmrEF benefit from medications that target the neurohormonal axes, a pathophysiological behavior that resembles that of HFrEF. Use of β-blockers, angiotensin-converting enzyme inhibitors, angiotensin receptor blockers, mineralocorticoid receptor antagonists, and sacubitril/valsartan is reasonable in patients with HFmrEF, whereas evidence is currently scarce for other therapies. In clinical practice, patients with HFmrEF are treated more like HFrEF patients, potentially because of history of systolic dysfunction that has partially recovered. Assessment of left ventricular systolic function with contemporary noninvasive modalities, e.g., echocardiographic strain imaging, is promising for the selection of patients with HFmrEF who will benefit from neurohormonal antagonists and other HFrEF-targeted therapies.

## 1. Introduction

Heart failure (HF) with mid-range ejection fraction (HFmrEF) is a relatively new entity, introduced by the European Society of Cardiology (ESC) in 2016, in an attempt to address the “gray zone” issue for patients with HF and left ventricular ejection fraction (LVEF) between 41% and 49% [1,2]. Currently HF is categorized into HF with reduced (LVEF ≤ 40%) ejection fraction (HFrEF), HFmrEF (LVEF 41% to 49%), and HF with preserved (LVEF ≥ 50%) ejection fraction (HFpEF) [2,3,4].

In this review, we briefly outline our current knowledge on the epidemiology, outcomes, and pathophysiology of HFmrEF, and discuss in detail the evidence on current treatment options for this group of patients with HF. Finally, we summarize gaps in knowledge and future perspectives.

## 2. Epidemiology

HFmrEF represents a sizeable proportion of patients with HF. In registries and clinical trials, the proportion of HFmrEF has ranged between 13–24% [5,6,7]. For example, in the trial of intensified versus standard medical therapy in Elderly patients with Congestive Heart Failure (TIME-CHF), which enrolled 622 patients with HF regardless of LVEF, 17% were classified as HFmrEF [5]. In a Swedish registry of 4942 patients, 18% had HFpEF, 19% HFmrEF, and 63% HFrEF at baseline [6].

In a pooled analysis from four community-based cohorts, older age, male sex, higher blood pressure, diabetes, and previous myocardial infarction all predicted incident HFmrEF, which accounted for 10% of new HF cases [8]. In the ESC HF Long-Term Registry, a multinational registry of patients with HF presenting in European and Mediterranean centers, among 9134 patients the HFmrEF group (24% of patients) had some common features with the HFrEF group, including age, gender, and ischemic etiology, but had less dilation of the left ventricle and left atrium [7]. Similarly, in a study of 5236 patients from Australia, the prevalence of most risk factors among HFmrEF patients was intermediate between that observed for patients with HFrEF and HFpEF [9].

## 3. Outcomes

In a pooled analysis from four community-based cohorts [8], mortality after the onset of HFmrEF was worse than that of HFpEF (50 versus 39 events per 1000 person-years, *p* = 0.02), and comparable to that of HFrEF (46 events per 1000 person-years, *p* = 0.78). In the ESC HF Long-Term Registry, patients with HFmrEF experienced a one-year mortality of 7.6%, a rate intermediate between that observed in HFrEF (8.8%) and HFpEF (6.3%) [7]. Of note, low systolic blood pressure and high heart rate were predictors for mortality in both HFrEF and HFmrEF [7]. However, data from Australia suggest that mortality does not differ significantly among the three groups, with 30-day mortality ranging between 1.2% and 1.7%, one-year between 13.7% and 16.5%, and three-year between 29.0% and 30.0% [9]. In contrast, one-year readmission rates were higher for HFpEF (45.4%), followed by HFmrEF (42.4%) and HFrEF (40.2%), largely due to non-HF readmissions [9]. Finally, a meta-analysis detected a slightly lower relative risk (0.90; 95% confidence interval 0.85–0.94; *p* < 0.001) for mortality among patients with HFmrEF vs. HFrEF, but no significant differences in terms of all-cause or HF hospitalization [10].

## 4. Pathophysiology

The pathophysiology of HFmrEF is incompletely understood. Mild left ventricular [11] systolic impairment may not adequately explain clinical manifestations, and invoking diastolic dysfunction may be an oversimplification [12]. Circulating biomarkers can provide insights into the degree of neurohormonal activation and potentially assist in individualized management [13]. N-terminal pro-B-type natriuretic peptide (NT-proBNP) levels are similarly elevated in HFrEF and HFmrEF and significantly higher compared to HFpEF [5]. On the other hand, some evidence suggests that the neuroendocrine profile of patients with HFmrEF is similar to that of HFpEF [14], as are factors limiting exercise tolerance [15]. In a study investigating biomarkers from different pathophysiologic domains in patients with acute HF, HFrEF was associated predominantly with cardiac stretch and HFpEF with cardiac inflammation, and HFmrEF with both stretch and inflammation [16]. Similarly, cardiac troponin values in HFmrEF patients are intermediate to those with HFrEF and HFpEF [17].

In all, the existing evidence suggests that HFmrEF is characterized by mixed pathophysiology. In addition, the trajectory of LV systolic function, i.e., whether a patient develops midrange LVEF as a result of worsening versus improving LVEF [12,18], and the etiology of HF are important [19]. In this line, a recent expert consensus focuses more on the pathophysiological mechanisms of HF rather than LVEF [19]. As a subset of patients with HFmrEF appears to have more intense neurohormonal activation, therapies that block the neurohormonal axes may work in these patients, resembling the effects seen in HFrEF. We discuss below the current evidence for therapies in HFmrEF.

## 5. Established Therapies

### 5.1. Beta Blockers

Beta blockers are a cornerstone of pharmacotherapy in HFrEF, as large randomized controlled trials with these agents have demonstrated beneficial effects on mortality and hospitalizations.

In an individual-level meta-analysis of 11 trials [20], β-blockers halved cardiovascular mortality in patients with LVEF 40–49% in sinus rhythm (hazard ratio (HR) 0.48, 95% confidence interval (CI) 0.24–0.97; *p* = 0.040), regardless of ischemic or nonischemic etiology. The benefits with β-blockers were similar to those observed in HFrEF and included reductions in both sudden death and HF-related death, albeit the number of events was small [20]. However, there was no effect on cardiovascular hospitalizations in the HFmrEF group [20]. Compared to placebo, β-blockers led to increases in LVEF regardless of rhythm (sinus or atrial fibrillation) in the HFmrEF group, with more pronounced benefit when the etiology was ischemic [20]. Outcomes in patients with HFmrEF in atrial fibrillation were not better with β-blockers; however, the number of events was too small to draw firm conclusions [20]. In line with these findings, a Japanese registry reported that among patients with chronic HF, β-blockers were associated with better clinical outcomes in both HFmrEF and HFrEF patients, including comparable reductions in mortality (HR 0.57, 95%CI 0.37–0.87, *p* = 0.010; and HR 0.59, 95%CI 0.40–0.87, *p* = 0.008, respectively), but not in HFpEF patients [21]. In contrast to the meta-analysis by Cleland et al. [20], data from the Swedish Heart Failure Registry suggest that the one-year mortality benefit seen at one year with β-blockers in patients with HFmrEF is restricted to those with underlying coronary artery disease; mortality was reduced in HFmrEF with CAD (HR 0.74, 95%CI 0.59–0.92) but not in HFmrEF without CAD (HR 0.99, 95%CI 0.78–1.26) [22]. Of note, in the same registry, angiotensin-converting enzyme inhibitors (ACEIs) and ARBs reduced the risk of death regardless of CAD [22].

Similar findings have been reported in acute settings. In a national registry from Portugal studying 9429 patients with acute coronary syndromes between 2010 and 2016, in-hospital mortality was 0.9%, 2.4%, and 11.4% among patients with pre-discharge LVEF <40%, 40–49%, and ≥50%, respectively. In-hospital β-blocker administration was associated with reduced mortality in the midrange and reduced LVEF groups [23].

In all, most evidence suggests a possible beneficial effect of β-blockers for short term and potentially long-term outcomes in patients with HFmrEF (Table 1). Of note, the evidence contrasts with the recent ESC guideline update, which recommends that treatment of HFmrEF patients should be based on the evidence for HFpEF, which does not recommend β-blocker therapy [21,23]. As a result, a patient with a baseline LVEF of 36% that improves to 41% after medical or device therapy would change category to HFmrEF and, per the guidelines, would have discontinued β-blockers [23].

### 5.2. Angiotensin-Converting Enzyme Inhibitors (ACEI) and Angiotensin Receptor Blockers (ARB)

The current guidelines recommend therapies for HFmrEF on the basis of the evidence for HFpEF rather than that for HFrEF [24], as data for HFmrEF come mostly from patients at the lower end of the LVEF spectrum in HFpEF studies [2,24]. However, in practice, angiotensin-converting enzyme inhibitors (ACEIs), angiotensin receptor blockers (ARBs), and mineralocorticoid receptor antagonists (MRAs), as well as β-blockers, are used widely in this group of patients, based on post hoc analyses showing benefits in HFmrEF patients similar to those with HFrEF [24].

Data from the Swedish HF registry suggest that ACEIs/ARBs are beneficial in HFmrEF. Among 42,061 patients, 21% had HFmrEF; in this subgroup, ACEIs/ARBs were associated with reduced risk of death irrespective of the presence or absence of CAD (HR 0.67, 95%CI 0.56–0.80; and HR 0.59, 95%CI 0.48–0.72, respectively) [22].

A considerable amount of data came from the CHARM trial program. In CHARM-PRESERVED, which looked at the effect of candesartan in patients with LVEF > 40%, candesartan marginally improved the primary composite of cardiovascular death or HF hospitalization (covariate adjusted HR 0.86, 95%CI 0.74–1.00, *p* = 0.051). It is important to point out, however, that the study population comprised of patients with LVEF > 40% in general and not strictly HFmrEF or HFpEF patients [25]. In a newer analysis of the CHARM data, patients with HFmrEF comprised 17% (*n* = 1322) of the study population and had baseline characteristics similar to those with HFrEF [26]. Candesartan reduced the primary outcome event rate compared to placebo in patients with HFmrEF (7.4 vs. 9.7 per 100 patient-years; HR 0.76, 95%CI 0.61–0.96; *p* = 0.02). Interestingly, a similarly benefit was shown in HFrEF (HR 0.82, 95%CI 0.75–0.91; *p* < 0.001), but not in HFpEF (HR 0.95, 95%CI 0.79–1.14; *p* = 0.57) [26]. Also, in the same analysis, candesartan reduced the rate of recurrent HF hospitalizations by half in HFmrEF (incidence rate ratio 0.48, 95%CI 0.33–0.70; *p* < 0.001).

In conclusion, ACEIs and ARBs seem to be safe and effective therapies for HFmrEF, but further studies focused on this specific population are needed to provide concrete and generalizable answers.

### 5.3. Mineralocorticoid Receptor Antagonists (MRAs)

There is a considerable amount of evidence that would make the use of MRAs reasonable in patients with HFmrEF (Table 2). TOPCAT (Treatment of Preserved Cardiac Function HF with an Aldosterone Antagonist), a pivotal trial in HFpEF, randomized patients with LVEF ≥ 45% to receive either spironolactone or placebo. Although the main study was neutral, a post hoc analysis reported that patients with LVEF at the lower end of the spectrum were more likely to benefit from spironolactone with respect to the primary composite (cardiovascular death, HF hospitalization, or aborted cardiac arrest) and HF hospitalization [27]. However, TOPCAT was underpowered to prove superiority in this subgroup of patients. Of note, spironolactone did not benefit patients with LVEF > 60% [27].

In line with these findings, the Japanese Cardiac Registry of HF reported that spironolactone at the time of discharge in patients with HFmrEF was associated with lower rates of the composite of all-cause death or HF rehospitalization over 2.2 years [30]. In a retrospective study from China, the one-year rate of death or HF rehospitalization was lower among patients receiving spironolactone 50 or 25 mg daily compared to untreated patients (21.3% vs. 34.5%, *p* = 0.014), without a difference between the high and low spironolactone dose groups (21.8% vs. 20.7%, respectively, *p* = 0.861) [28].

In a recent meta-analysis of 11 randomized clinical trials that investigated the efficacy and safety of spironolactone in patients with HFmrEF and HFpEF [29], spironolactone was associated with a reduction in hospitalizations (OR = 0.84; 95%CI = 0.73–0.95; *p* = 0.006), B-type natriuretic peptide (BNP) levels (mean difference 44.80 pg/mL; 95%CI + 73.44–16.17; *p* = 0.002) and myocardial fibrosis, and improved New York Heart Association (NYHA) class (OR for improvement 0.35; 95%CI, 0.19–0.66; *p* = 0.001). The only noteworthy adverse effects were hyperkalemia and gynecomastia.

### 5.4. Sacubitril/Valsartan

Sacubitril/valsartan has been proven to decrease mortality and HF hospitalizations in patients with HFrEF, but its efficacy in HFmrEF remains unclear.

Following the successful PARADIGM-HF trial in HFrEF, PARAGON-HF studied the effect of angiotensin receptor neprilysin inhibition in patients with HFpEF [31], randomizing 4822 patients with symptomatic HF, LVEF ≥ 45%, elevated natriuretic peptides, and structural heart disease into sacubitril–valsartan or valsartan. Sacubitril/valsartan did not significantly lower the composite of total hospitalizations for HF and death from cardiovascular causes, despite modest improvements in NYHA class and less decline in renal function [31]. However, in a planned subgroup analysis, those with LVEF below the median (57%), but not those with LVEF above the median, benefited from sacubitril/valsartan. The observed rate ratio in the below-median subgroup (0.78; 95%CI 0.64–0.95) was similar to that observed in the HFrEF-focused PARADIGM-HF (HR 0.80; 95%CI 0.73–0.87) [31]. In a recent meta-analysis of six studies with over 5500 patients, compared with ACEIs and ARBs, sacubitril-valsartan reduced hospitalizations for HF (risk ratio 0.84; 95%CI 0.77–0.91; *p* < 0.001) and improved NYHA class (risk ratio 1.25; 95%CI 1.10–1.43; *p* = 0.001) in HFmrEF and HFpEF patients [32].

## 6. Other Therapeutics

### 6.1. Ivabradine

Heart rate is another potentially modifiable factor that has been linked to worse outcomes in patients with HF, including those with HFmrEF [33]. In a recent report, presence of atrial fibrillation and elevated heart rates were independently correlated with impaired peak VO_2_ and were associated with adverse prognosis in patients with HFmrEF [34]. Ivabradine lowers the heart rate though I*_f_* current inhibition in the sinoatrial node; when a heart rate of <70 bpm is achieved, there is a well-documented benefit on outcomes, primarily hospitalizations, among patients with HFrEF [35], and lower rates of coronary events among patients with coronary artery disease and reduced LVEF [36]. Hypothesizing that these findings could be extrapolated to HFmrEF, a Chinese retrospective study of 197 hospitalized patients with HFmrEF reported that a HR < 70 bpm was associated with a lower risk of the composite of HF readmission or all-cause death and better quality of life, particularly among those prescribed with β-blockers [33].

### 6.2. Ranolazine

Ranolazine is an antianginal agent that acts on the late sodium (I*_na_*) current. In patients with HF, by acting on the I*_na_* current, ranolazine has the potential to mechanistically reduce the influx of Ca^++^ within the myocyte and reduce its deleterious effects, i.e., diastolic dysfunction, microvascular compression, and eventually worsening of LV function [37]. In an open-label study in HF, ranolazine was associated with a significant increase in LVEF after 2 years, regardless of baseline LVEF [38]. This increase was more prominent in patients with LVEF ≥ 40%, although no midrange-specific data were available [38].

### 6.3. Digoxin

In a retrospective analysis of the Digitalis Investigation Group (DIG) trial evaluating the effect of digoxin in patients with HFmrEF [39], digoxin reduced the composite of cardiovascular death or HF hospitalization, mainly driven by the reduction of HF hospitalizations. The digoxin/placebo hazard ratio for HF hospitalization was 0.71 (95%CI 0.65–0.77) for HFrEF, 0.80 (95%CI 0.63–1.03) for HFmrEF, and 0.85 (95%CI 0.62–1.17) for HFpEF, while the digoxin/placebo HR for the composite of HF death or HF hospitalization was 0.74 (95%CI 0.68–0.81), 0.83 (95%CI 0.66–1.05), and 0.88 (95%CI 0.65–1.19), respectively [39]. Interestingly, digoxin had the strongest effect on HF hospitalization in patients with HFrEF, an intermediate effect in HFmrEF, and the smallest effect in HFpEF [39]. This pattern is consistent with other therapies that demonstrated benefit primarily in HFrEF.

### 6.4. Antidiabetic Medications

In the SwedeHF (Swedish Heart Failure) registry, among >30,000 patients, the prevalence of type 2 diabetes (T2D) was similar across HF subgroups (HFpEF, 25%; HFmrEF and HFrEF, 24%). Interestingly, T2D was a significant mortality prognosticator across the LVEF spectrum, with its effect being more prominent in the HFmrEF and HFrEF groups where T2D increased mortality risk by 50% compared to 30% in HFpEF group [40].

The sodium/glucose cotransporter 2 (SGLT2) inhibitors are the first class of antidiabetics that improve HF outcomes in patients with HFrEF, including HF hospitalizations, regardless of diabetic status [41]. However, there are no data yet specifically for the HFmrEF population.

### 6.5. Levosimendan

A recent meta-analysis of nine randomized controlled trials suggested that IV levosimendan can reduce BNP level and increase LVEF in patients with advanced HF, including patients at the higher end of reduced LVEF [42]. Whether these findings can be extrapolated to patients with HFmrEF needs further investigation.

## 7. Challenges in HFmrEF Management 

### 7.1. Atrial Fibrillation in Patients with HFmrEF

Atrial fibrillation is common across the LVEF spectrum [43]. In a report from the SwedeHF registry investigating the role of AF in patients with HFpEF, HFmrEF, and HFrEF, although the prevalence of AF increased as LVEF increased, the clinical characteristics of patients were almost identical among the three groups [43]. AF was also linked to an increase in the number of deaths, HF hospitalizations, and strokes or transient ischemic attacks in all LVEF groups [43]. Similar findings were reported among 15,000 patients in the HF Long-Term Registry of the European Society of Cardiology [44]. The prevalence of AF was higher with increasing LVEF (27% in HFrEF, 29% in HFmrEF, and 39% in HFpEF) and AF was associated with worse outcomes (combined all-cause mortality and HF hospitalization) in HFpEF (HR = 1.36, 95%CI 1.15–1.62, *p* < 0.001) and HFmrEF (HR = 1.30, 95%CI 1.06–1.61, *p* =  0.014), but not in HFrEF (HR = 0.96, 95%CI 0.84–1.09, *p* = 0.502) [44]. These findings were confirmed in a prospective study from China [45]. Finally, in a retrospective study of 128 patients with HFmrEF, AF was associated with worsening exercise capacity, impaired peak VO_2_, which is a surrogate for exercise capacity in HF patients, and adverse prognosis [34].

### 7.2. Acute HFmrEF and Treatment

Hospitalized HFmrEF patients represent a demographically and clinically diverse group of patients that shares similarities with both HFrEF and HFpEF patients [46]. As a result, the optimal treatment of patients with acute HFmrEF remains unclear. A few studies have addressed this issue.

The Korean Acute Heart Failure (KorAHF) registry is a prospective multicenter cohort of hospitalized acute HF patients in Korea [47]. From a total of 5374 patients, 58% had HFrEF, 16% had HFmrEF, and 25% had HFpEF [47]. HFmrEF patients seemed to have intermediate clinical profiles between HFrEF and HFpEF. Lower LVEF was associated with worse short-term outcomes and all-cause in-hospital mortality (7.1%, 3.6%, and 3.0%, for HFrEF, HFmrEF and HFpEF, respectively). Importantly, the three-year all-cause mortality was 37.6% and comparable among the three LVEF groups.

In the international Acute Heart Failure Global Registry of Standard Treatment (ALARM-HF), most patients with HFmrEF (93.3%) received intravenous loop diuretics, while 47.5% received intravenous vasodilators, and 30.0% inotropes [46]. These numbers were similar to HFrEF and HFpEF for loop diuretics, but higher vs. the other three groups for vasodilators (*p* = 0.030), and lower vs. HFrEF for inotropes (*p* < 0.001) [46]. Of hospitalized HFmrEF patients, 51.7% also received β-blockers, 80.7% ACEIs or ARBs, and 26.9% MRAs [46]. Mortality at 30 days was 9.4% among HFmrEF patients, significantly lower compared to HFrEF (HR 0.64; 95%CI 0.42–0.96, *p* = 0.033) but not to HFpEF (HR 1.03; 95%CI 0.60–1.74, *p* = 0.923) [46].

### 7.3. Cardiac Resynchronization Therapy (CRT)

Data on CRT as de novo therapy for patients with HFmrEF are lacking, as intraventricular dys-synchrony is a function of LVEF, and gross dys-synchrony is uncommon with relatively preserved LVEF. However, in recent reports, a substantial number of CRT recipients appear to have entered a chronic phase of “recovered” HFmrEF or HFpEF [48]. It is unclear whether continuing CRT would be beneficial for these patients, e.g., whether replacing the generator or problematic leads is indicated.

### 7.4. What Is Happening in the Real World?

How do providers handle this special group of patients, considering the lack of specific data or guidelines? In the Chronic Heart failure ESC-guideline based Cardiology Practice Quality project (CHECK-HF) registry, which enrolled over 10,000 HF patients in the Netherlands, the prescription patterns for guideline-directed medical therapy did not significantly differ between the HFrEF and HFmrEF groups, with 83.2% of HFmrEF patients receiving an ACEI or ARB, 81.0% a β-blocker, and 56.4% an MRA [49]. However, clear guidelines are necessary for the optimal treatment of this group of patients.

In the American College of Cardiology Practice Innovation and Clinical Excellence (PINNACLE) Registry [50], which included HF patients from 2008 to 2016, patients with HFmrEF in everyday practice were treated with guideline directed medical therapy, with use of ACEIs/ARBs, β-blockers, and diuretics matching the patterns seen in HFrEF rather than HFpEF. This could be potentially due to clinicians assuming that HFmrEF constitutes HFrEF with only partially recovered LVEF, or because patients are already treated with these medications for other medical conditions, e.g., hypertension or previous CAD [51].

## 8. Future Perspectives

There is a need for reliable studies looking at the efficacy of established agents in patients with HFmrEF. Emerging therapeutic agents for HFrEF could also be efficacious for patients with HFmrEF.

### 8.1. Vericiguat

Vericiguat increases soluble guanylate cyclase activity. By stimulating production of cyclic guanosine monophosphate, vericiguat can improve myocardial and vascular function. In VICTORIA, a double-blind randomized trial, 5050 patients with LVEF < 45% and recent HF hospitalization or intravenous diuretic use were randomly assigned to either vericiguat or placebo [52]. The primary endpoint of cardiovascular death or HF hospitalization occurred in 35.5% of the active vs. 38.5% of the placebo group (HR 0.90, 95%CI 0.82–0.98; *p* = 0.019). However, there was no signal of benefit in the small subgroup of patients (14.3% of the trial population) with LVEF ≥ 40 (HR 1.05, 95%CI 0.81–1.36). Importantly, in a post hoc analysis, a reduction in cardiovascular death and hospitalization for HF was observed with vericiguat for the subgroup of patients with lower NT-proBNP (≤8000 pg/mL), indicating that patients who are too sick may not benefit from vericiguat and that earlier initiation of this agent may be more efficacious [53]. Whether this applies to HFmrEF patients remains to be investigated.

### 8.2. Tolvaptan

Tolvaptan is a vasopressin V2 receptor antagonist approved for the treatment of fluid retention in HF patients in Japan and for hyponatremia in the United States [54]. Previous studies have shown that tolvaptan is safe and effective in HFrEF patients, but data for HFpEF and HFmrEF remain inconclusive [54]. In a subgroup analysis of the post-marketing surveillance SMILE study of 1741 patients, tolvaptan led to significant body weight reductions and increases in 24-h urine volume, as well as improvement in congestive symptoms over the 14-day treatment period, regardless of LVEF [54]. More data is needed before conclusions can be drawn for HFmrEF.

### 8.3. Exercise Training Programs

While rest was the widely accepted recommendation for HF patients for years, recent data showed that physical inactivity may play a key role in symptomatic worsening and poor outcomes. In the HF Adherence and Retention Trial (HART), physical inactivity was associated with almost double all-cause and cardiovascular mortality in patients with NYHA II/III HF across the LVEF spectrum, whereas even modest exercise was linked to improved survival [55]. Exercise training is strongly recommended for patients with NYHA II-III HF as it is proven to relieve symptoms, improve exercise capacity, quality of life, and reduce disability and hospitalization rates [56].

In a meta-analysis of 14 randomized controlled trials, aerobic training improved LVEF with an increase of 2.59% [57], while another systematic review confirmed that exercise training has beneficial effects on LV remodeling in clinically stable ischemic patients when exercise intervention starts early after a myocardial infarction [58]. Even though these studies were not designed to assess the efficacy of physical activity specifically in HFmrEF, it is reasonable to extrapolate these results to HFmrEF, especially given the absence of any harm signal, until more definitive data become available.

## 9. Is LVEF an Adequate Marker to Guide Therapy for HF?

Is LVEF an adequate indicator of systolic dysfunction—and consequently a reliable means of phenotyping HF into subtypes, with the goal of therapy selection? The answer is not straightforward. An impaired LVEF is a highly specific marker of systolic dysfunction, but not a sensitive one. That is, absence of a clearly impaired LVEF does not guarantee good systolic function, i.e., a physiology that would not benefit from anti-neurohormonal therapies. For this reason, most HFrEF therapies appear to offer some benefit to patients with HFmrEF, as this is a mixed group of patients that most certainly includes patients with more intense neurohormonal activation as a result of systolic dysfunction not immediately evident by examining LVEF. More refined tools, e.g., echocardiographic strain imaging, have shown that systolic dysfunction is common among HFmrEF and even HFpEF patients [59,60,61,62].

LVEF is a fluid marker. In SwedeHF, among patients with ≥2 LVEF assessments (median: 1.4 years, interquartile range 0.5 to 3.0 years), 21% and 18% of HFpEF patients transitioned to HFmrEF and HFrEF, respectively; 37% and 25% of HFmrEF transitioned to HFrEF and HFpEF, respectively; and 16% and 10% of HFrEF transitioned to HFmrEF and HFpEF, respectively [6].

Nevertheless, LVEF is still widely used to categorize HF. However, newer methods to assess LV function across the HF spectrum are integrated in clinical research and practice [19,56,63]. The most promising non-invasive method is echocardiographic strain (deformation) imaging [64]. In the Strain for Risk Assessment and Therapeutic Strategies in Patients with Acute Heart Failure registry, use of β-blockers was associated with reduced mortality in patients with global longitudinal strain (GLS) <14% among 692 patients with HFmrEF (adjusted HR 0.64; 95%CI 0.46–0.90; *p* = 0.010) and 1227 patients with HFpEF (adjusted HR 0.57; 95%CI 0.41–0.80; *p* = 0.001), but not in those with GLS ≥ 14% [61].

Importantly, even for HFrEF and HFpEF, there is much phenotypic similarity, e.g., in LV size and wall thickness. HFmrEF is even more heterogenous, as midrange LVEF may result from HFrEF with partially recovered LVEF, HFpEF with declined LVEF, or de novo HF presentation. On top of these overlapping entities, LVEF has an intra-observer and interobserver variability that often exceeds 5% [65]. Etiology may also play a role in therapy selection for HFmrEF, as CAD, hypertensive heart disease, and nonischemic cardiomyopathy need different approaches. Finally, machine learning and pheno-mapping, which have been applied to HFpEF for the identification of prognostic subgroups with potentially different therapeutic needs [66], could be used to identify HFmrEF subgroups.

## 10. Conclusions

The “HFmrEF” category has generated both interest and controversy. The available data suggest that patients with HFmrEF have an intermediate phenotype between HFpEF and HFrEF in terms of baseline characteristics, outcomes, and prognosis, slightly resembling more that of a HFrEF patient than of HFpEF. Of note, studies have shown that a considerable number of patients transition to either HFrEF or HFpEF while on treatment. Studies targeting this population specifically are needed to shed light on the intricacies of the pathophysiology and phenotype of these patients, hopefully leading to more personalized treatment plans.

## Figures and Tables

**Table 1:** Key Studies with β-Blockers in Patients with heart failure (HF) with mid-range ejection fraction (HFmrEF).

Type of Study	Reference	HFmrEF Population	Findings
Metanalysis of randomized controlled trials	Cleland et al., 2018 [20]	721 patients with LVEF 40–49% (575 in sinus rhythm, 146 in AF); median follow-up was 1.3 years (IQR 0.8–1.9) for the entire study	Beta-blockers were associated with decreased cardiovascular (adjusted HR 0.48, 95%CI 0.24–0.97) and all-cause (adjusted HR 0.59, 95%CI 0.34–1.03) mortality among patients with LVEF 40–49% in sinus rhythm, but not among those with AF. There was no effect on cardiovascular hospitalizations.
Multicenter prospective registry, Japan	Tsuji et al., 2017 [21]	596 patients with LVEF 40–49%, age 69 ± 12 years, 28.2% women, followed up to 3 years	Use of beta-blockers was associated with reduced mortality in HFmrEF patients (adjusted HR 0.57, 95%CI 0.37–0.87; *p* = 0.010).
Retrospective study, nationwide registry, Portugal	Montenegro et al., 2019 [23]	1926 patients with acute coronary syndrome and EF 40–49%	In-hospital β-blockers were associated with reduced in-hospital mortality (adjusted HR 0.3, 95%CI 0.1–0.6; *p* = 0.003) in these patients; however, number of events was small.
Nationwide registry, Sweden	Koh et al., 2017 [22]	Of 42061 patients 21% had HFmrEF, mean age was 74 ± 12 years, women 39%	53% of the HFmrEF group had CAD, which modified the association between β-blocker and 1-year mortality, which was reduced in HFmrEF with CAD (HR up to 1 year 0.74, 95%CI: 0.59–0.92) but not in HFmrEF without CAD (HR 0.99, 95%CI: 0.78–1.26).

AF: atrial fibrillation; CAD: Coronary artery disease; CI: confidence interval; HR: hazard ratio; IQR: interquartile range; LVEF: left ventricular ejection fraction.

**Table 2:** Key Studies with Mineralocorticoid Receptor Antagonists in Patients with HFmrEF.

Type of Study	Reference	HFmrEF Population	Findings
Post-hoc analysis of randomized clinical trial	Solomon et al., 2016 [27]	520 patients (197 in the Americas) with LVEF 45–50% randomized to placebo or spironolactone, followed for a median of 3.4 years	Patients in this group benefited more from spironolactone; HR for primary endpoint (death or HF hospitalization) was 0.55 (95%CI 0.33–0.91); and for CV death 0.46 (95%CI 0.23–0.94)
Retrospective cohort, China	Xin et al., 2019 [28]	279 HFmrEF patients divided into 3 groups: high-dose (50 mg daily), low-dose (25 mg daily) and no spironolactone	Patients on spironolactone had lower rate of 1-year death or HF rehospitalization vs. untreated (21.3% vs. 34.5%, *p* = 0.014); no difference between high vs. low dose (21.8% vs. 20.7%, *p* = 0.861)
Meta-analysis of randomized clinical trials	Xiang et al., 2019 [29]	4539 HFmrEF and HFpEF patients; 375 had myocardial disease, 108 hypertension, and 4056 multiple or unclear etiology; 770 patients with LVEF ≥ 50% and 3769 patients with LVEF ≥ 40% or ≥45%	Spironolactone reduced readmission (odds ratio 0.84; 95%CI 0.73–0.95; *p* = 0.006) and PICP levels (mean difference, −27.04 ng/mL; 95%CI, −40.77 to −13.32; *p* < 0.001) in patients with HFmrEF and HFpEF

CV: cardiovascular; HF: heart failure; HR: hazard ratio; LVEF: left ventricular ejection fraction; PICP: procollagen type I C-terminal pro-peptide.

## References

[1] Lam C.S., Solomon S.D. (2014). The middle child in heart failure: Heart failure with mid-range ejection fraction (40–50%). Eur. J. Heart Fail..

[2] Ponikowski P., Voors A.A., Anker S.D., Bueno H., Cleland J.G.F., Coats A.J.S., Falk V., Gonzalez-Juanatey J.R., Harjola V.P., Jankowska E.A. (2016). 2016 ESC Guidelines for the diagnosis and treatment of acute and chronic heart failure: The Task Force for the diagnosis and treatment of acute and chronic heart failure of the European Society of Cardiology (ESC) Developed with the special contribution of the Heart Failure Association (HFA) of the ESC. Eur. Heart J..

[3] Hsu J.J., Ziaeian B., Fonarow G.C. (2017). Heart Failure With Mid-Range (Borderline) Ejection Fraction: Clinical Implications and Future Directions. JACC Heart Fail..

[4] Srivastava P.K., Hsu J.J., Ziaeian B., Fonarow G.C. (2020). Heart Failure With Mid-range Ejection Fraction. Curr. Heart Fail. Rep..

[5] Rickenbacher P., Kaufmann B.A., Maeder M.T., Bernheim A., Goetschalckx K., Pfister O., Pfisterer M., Brunner-La Rocca H.P., Investigators T.-C. (2017). Heart failure with mid-range ejection fraction: A distinct clinical entity? Insights from the Trial of Intensified versus standard Medical therapy in Elderly patients with Congestive Heart Failure (TIME-CHF). Eur. J. Heart Fail..

[6] Savarese G., Vedin O., D’Amario D., Uijl A., Dahlstrom U., Rosano G., Lam C.S.P., Lund L.H. (2019). Prevalence and Prognostic Implications of Longitudinal Ejection Fraction Change in Heart Failure. JACC Heart Fail..

[7] Chioncel O., Lainscak M., Seferovic P.M., Anker S.D., Crespo-Leiro M.G., Harjola V.P., Parissis J., Laroche C., Piepoli M.F., Fonseca C. (2017). Epidemiology and one-year outcomes in patients with chronic heart failure and preserved, mid-range and reduced ejection fraction: An analysis of the ESC Heart Failure Long-Term Registry. Eur. J. Heart Fail..

[8] Bhambhani V., Kizer J.R., Lima J.A.C., van der Harst P., Bahrami H., Nayor M., de Filippi C.R., Enserro D., Blaha M.J., Cushman M. (2018). Predictors and outcomes of heart failure with mid-range ejection fraction. Eur. J. Heart Fail..

[9] Wang N., Hales S., Barin E., Tofler G. (2018). Characteristics and outcome for heart failure patients with mid-range ejection fraction. J. Cardiovasc. Med. (Hagerstown).

[10] Altaie S., Khalife W. (2018). The prognosis of mid-range ejection fraction heart failure: A systematic review and meta-analysis. ESC Heart Fail..

[11] Zinman B., Wanner C., Lachin J.M., Fitchett D., Bluhmki E., Hantel S., Mattheus M., Devins T., Johansen O.E., Woerle H.J. (2015). Empagliflozin, Cardiovascular Outcomes, and Mortality in Type 2 Diabetes. N. Engl. J. Med..

[12] Rastogi A., Novak E., Platts A.E., Mann D.L. (2017). Epidemiology, pathophysiology and clinical outcomes for heart failure patients with a mid-range ejection fraction. Eur. J. Heart Fail..

[13] Unkovic P., Basuray A. (2018). Heart Failure with Recovered EF and Heart Failure with Mid-Range EF: Current Recommendations and Controversies. Curr. Treat. Options Cardiovasc. Med..

[14] Vergaro G., Aimo A., Prontera C., Ghionzoli N., Arzilli C., Zyw L., Taddei C., Gabutti A., Poletti R., Giannoni A. (2019). Sympathetic and renin-angiotensin-aldosterone system activation in heart failure with preserved, mid-range and reduced ejection fraction. Int. J. Cardiol..

[15] Pugliese N.R., Fabiani I., Santini C., Rovai I., Pedrinelli R., Natali A., Dini F.L. (2019). Value of combined cardiopulmonary and echocardiography stress test to characterize the haemodynamic and metabolic responses of patients with heart failure and mid-range ejection fraction. Eur. Heart J. Cardiovasc. Imaging.

[16] Tromp J., Khan M.A.F., Mentz R.J., O’Connor C.M., Metra M., Dittrich H.C., Ponikowski P., Teerlink J.R., Cotter G., Davison B. (2017). Biomarker Profiles of Acute Heart Failure Patients With a Mid-Range Ejection Fraction. JACC Heart Fail..

[17] Gohar A., Chong J.P.C., Liew O.W., den Ruijter H., de Kleijn D.P.V., Sim D., Yeo D.P.S., Ong H.Y., Jaufeerally F., Leong G.K.T. (2017). The prognostic value of highly sensitive cardiac troponin assays for adverse events in men and women with stable heart failure and a preserved vs. reduced ejection fraction. Eur. J. Heart Fail..

[18] Kalogeropoulos A.P., Fonarow G.C., Georgiopoulou V., Burkman G., Siwamogsatham S., Patel A., Li S., Papadimitriou L., Butler J. (2016). Characteristics and Outcomes of Adult Outpatients With Heart Failure and Improved or Recovered Ejection Fraction. JAMA Cardiol..

[19] Triposkiadis F., Butler J., Abboud F.M., Armstrong P.W., Adamopoulos S., Atherton J.J., Backs J., Bauersachs J., Burkhoff D., Bonow R.O. (2019). The continuous heart failure spectrum: Moving beyond an ejection fraction classification. Eur. Heart J..

[20] Cleland J.G.F., Bunting K.V., Flather M.D., Altman D.G., Holmes J., Coats A.J.S., Manzano L., McMurray J.J.V., Ruschitzka F., van Veldhuisen D.J. (2018). Beta-blockers for heart failure with reduced, mid-range, and preserved ejection fraction: An individual patient-level analysis of double-blind randomized trials. Eur. Heart J..

[21] Tsuji K., Sakata Y., Nochioka K., Miura M., Yamauchi T., Onose T., Abe R., Oikawa T., Kasahara S., Sato M. (2017). Characterization of heart failure patients with mid-range left ventricular ejection fraction-a report from the CHART-2 Study. Eur. J. Heart Fail..

[22] Koh A.S., Tay W.T., Teng T.H.K., Vedin O., Benson L., Dahlstrom U., Savarese G., Lam C.S.P., Lund L.H. (2017). A comprehensive population-based characterization of heart failure with mid-range ejection fraction. Eur. J. Heart Fail..

[23] Montenegro Sa F., Carvalho R., Ruivo C., Santos L.G., Antunes A., Soares F., Belo A., Morais J., Portuguese Registry of Acute Coronary Syndrome Investigators (2019). Beta-blockers for post-acute coronary syndrome mid-range ejection fraction: A nationwide retrospective study. Eur. Heart J. Acute Cardiovasc. Care.

[24] Lopatin Y. (2018). Heart Failure with Mid-Range Ejection Fraction and How to Treat It. Card Fail. Rev..

[25] Yusuf S., Pfeffer M.A., Swedberg K., Granger C.B., Held P., McMurray J.J., Michelson E.L., Olofsson B., Ostergren J., Investigators C. (2003). Effects of candesartan in patients with chronic heart failure and preserved left-ventricular ejection fraction: The CHARM-Preserved Trial. Lancet.

[26] Lund L.H., Claggett B., Liu J., Lam C.S., Jhund P.S., Rosano G.M., Swedberg K., Yusuf S., Granger C.B., Pfeffer M.A. (2018). Heart failure with mid-range ejection fraction in CHARM: Characteristics, outcomes and effect of candesartan across the entire ejection fraction spectrum. Eur. J. Heart Fail..

[27] Solomon S.D., Claggett B., Lewis E.F., Desai A., Anand I., Sweitzer N.K., O’Meara E., Shah S.J., McKinlay S., Fleg J.L. (2016). Influence of ejection fraction on outcomes and efficacy of spironolactone in patients with heart failure with preserved ejection fraction. Eur. Heart J..

[28] Xin Y.G., Chen X., Zhao Y.N., Hu J., Sun Y., Hu W.Y. (2019). Outcomes of spironolactone treatment in patients in Northeast China suffering from heart failure with mid-range ejection fraction. Curr. Med. Res. Opin..

[29] Xiang Y., Shi W., Li Z., Yang Y., Wang S.Y., Xiang R., Feng P., Wen L., Huang W. (2019). Efficacy and safety of spironolactone in the heart failure with mid-range ejection fraction and heart failure with preserved ejection fraction: A meta-analysis of randomized clinical trials. Medicine.

[30] Enzan N., Matsushima S., Ide T., Kaku H., Higo T., Tsuchihashi-Makaya M., Tsutsui H. (2020). Spironolactone use is associated with improved outcomes in heart failure with mid-range ejection fraction. ESC Heart Fail..

[31] Solomon S.D., McMurray J.J.V., Anand I.S., Ge J., Lam C.S.P., Maggioni A.P., Martinez F., Packer M., Pfeffer M.A., Pieske B. (2019). Angiotensin-Neprilysin Inhibition in Heart Failure with Preserved Ejection Fraction. N. Engl. J. Med..

[32] Nie D., Xiong B., Qian J., Rong S., Yao Y., Huang J. (2020). The Effect of Sacubitril-Valsartan in Heart Failure Patients With Mid-Range and Preserved Ejection Fraction: A Meta-Analysis. Heart Lung Circ..

[33] Xin Y., Chen X., Zhao Y., Hu W. (2019). The impact of heart rate on patients diagnosed with heart failure with mid-range ejection fraction. Anatol. J. Cardiol..

[34] Sato Y., Yoshihisa A., Kimishima Y., Yokokawa T., Abe S., Shimizu T., Misaka T., Yamada S., Sato T., Kaneshiro T. (2019). Atrial fibrillation is associated with impaired exercise capacity and adverse prognosis in patients with heart failure with mid-range ejection fraction. Eur. J. Prev. Cardiol..

[35] Swedberg K., Komajda M., Bohm M., Borer J.S., Ford I., Dubost-Brama A., Lerebours G., Tavazzi L., Investigators S. (2010). Ivabradine and outcomes in chronic heart failure (SHIFT): A randomised placebo-controlled study. Lancet.

[36] Fox K., Ford I., Steg P.G., Tendera M., Ferrari R., Investigators B. (2008). Ivabradine for patients with stable coronary artery disease and left-ventricular systolic dysfunction (BEAUTIFUL): A randomised, double-blind, placebo-controlled trial. Lancet.

[37] Belardinelli L., Shryock J.C., Fraser H. (2006). Inhibition of the late sodium current as a potential cardioprotective principle: Effects of the late sodium current inhibitor ranolazine. Heart.

[38] Murray G.L., Colombo J. (2014). Ranolazine preserves and improves left ventricular ejection fraction and autonomic measures when added to guideline-driven therapy in chronic heart failure. Heart Int..

[39] Abdul-Rahim A.H., Shen L., Rush C.J., Jhund P.S., Lees K.R., McMurray J.J.V., Collaborators V.I.-H.F. (2018). Effect of digoxin in patients with heart failure and mid-range (borderline) left ventricular ejection fraction. Eur. J. Heart Fail..

[40] Johansson I., Dahlstrom U., Edner M., Nasman P., Ryden L., Norhammar A. (2018). Type 2 diabetes and heart failure: Characteristics and prognosis in preserved, mid-range and reduced ventricular function. Diab. Vasc. Dis. Res..

[41] Kumar K., Kheiri B., Simpson T.F., Osman M., Rahmouni H. (2020). Sodium-Glucose Cotransporter-2 Inhibitors in Heart Failure: A Meta-Analysis of Randomized Clinical Trials. Am. J. Med..

[42] Cui D., Liao Y., Li G., Chen Y. (2020). Levosimendan Can Improve the Level of B-Type Natriuretic Peptide and the Left Ventricular Ejection Fraction of Patients with Advanced Heart Failure: A Meta-analysis of Randomized Controlled Trials. Am. J. Cardiovasc. Drugs.

[43] Sartipy U., Dahlstrom U., Fu M., Lund L.H. (2017). Atrial Fibrillation in Heart Failure With Preserved, Mid-Range, and Reduced Ejection Fraction. JACC Heart Fail..

[44] Zafrir B., Lund L.H., Laroche C., Ruschitzka F., Crespo-Leiro M.G., Coats A.J.S., Anker S.D., Filippatos G., Seferovic P.M., Maggioni A.P. (2018). Prognostic implications of atrial fibrillation in heart failure with reduced, mid-range, and preserved ejection fraction: A report from 14 964 patients in the European Society of Cardiology Heart Failure Long-Term Registry. Eur. Heart J..

[45] Xu H.X., Zhu Y.M., Hua Y., Huang Y.H., Lu Q. (2020). Association between atrial fibrillation and heart failure with different ejection fraction categories and its influence on outcomes. Acta Cardiol..

[46] Farmakis D., Simitsis P., Bistola V., Triposkiadis F., Ikonomidis I., Katsanos S., Bakosis G., Hatziagelaki E., Lekakis J., Mebazaa A. (2017). Acute heart failure with mid-range left ventricular ejection fraction: Clinical profile, in-hospital management, and short-term outcome. Clin. Res. Cardiol..

[47] Cho J.H., Choe W.S., Cho H.J., Lee H.Y., Jang J., Lee S.E., Choi J.O., Jeon E.S., Kim M.S., Hwang K.K. (2019). Comparison of Characteristics and 3-Year Outcomes in Patients With Acute Heart Failure With Preserved, Mid-Range, and Reduced Ejection Fraction. Circ. J..

[48] Abe S., Yoshihisa A., Ichijo Y., Sato Y., Kanno Y., Takiguchi M., Yokokawa T., Misaka T., Sato T., Oikawa M. (2020). Recovered Left Ventricular Ejection Fraction and Its Prognostic Impacts in Hospitalized Heart Failure Patients with Reduced Ejection Fraction. Int. Heart J..

[49] Brunner-La Rocca H.P., Linssen G.C., Smeele F.J., van Drimmelen A.A., Schaafsma H.J., Westendorp P.H., Rademaker P.C., van de Kamp H.J., Hoes A.W., Brugts J.J. (2019). Contemporary Drug Treatment of Chronic Heart Failure With Reduced Ejection Fraction: The CHECK-HF Registry. JACC Heart Fail..

[50] Ezekowitz J.A., McAlister F.A. (2009). Aldosterone blockade and left ventricular dysfunction: A systematic review of randomized clinical trials. Eur. Heart J..

[51] Ibrahim N.E., Song Y., Cannon C.P., Doros G., Russo P., Ponirakis A., Alexanian C., Januzzi J.L. (2019). Heart failure with mid-range ejection fraction: Characterization of patients from the PINNACLE Registry(R). ESC Heart Fail..

[52] Armstrong P.W., Pieske B., Anstrom K.J., Ezekowitz J., Hernandez A.F., Butler J., Lam C.S.P., Ponikowski P., Voors A.A., Jia G. (2020). Vericiguat in Patients with Heart Failure and Reduced Ejection Fraction. N. Engl. J. Med..

[53] Ezekowitz J.A., O’Connor C.M., Troughton R.W., Alemayehu W.G., Westerhout C.M., Voors A.A., Butler J., Lam C.S.P., Ponikowski P., Emdin M. (2020). N-Terminal Pro-B-Type Natriuretic Peptide and Clinical Outcomes: Vericiguat Heart Failure With Reduced Ejection Fraction Study. JACC Heart Fail..

[54] Kinugawa K., Sato N., Inomata T., Yasuda M., Shimakawa T., Fukuta Y. (2019). A Prospective, Multicenter, Post-Marketing Surveillance Study to Evaluate the Safety and Effectiveness of Tolvaptan in Patients with Reduced, Preserved, and Mid-Range Ejection Fraction Heart Failure. Int. Heart J..

[55] Doukky R., Mangla A., Ibrahim Z., Poulin M.F., Avery E., Collado F.M., Kaplan J., Richardson D., Powell L.H. (2016). Impact of Physical Inactivity on Mortality in Patients With Heart Failure. Am. J. Cardiol..

[56] Katsi V., Georgiopoulos G., Laina A., Koutli E., Parissis J., Tsioufis C., Nihoyannopoulos P., Tousoulis D. (2017). Left ventricular ejection fraction as therapeutic target: Is it the ideal marker?. Heart Fail. Rev..

[57] Haykowsky M.J., Liang Y., Pechter D., Jones L.W., McAlister F.A., Clark A.M. (2007). A meta-analysis of the effect of exercise training on left ventricular remodeling in heart failure patients: The benefit depends on the type of training performed. J. Am. Coll. Cardiol..

[58] Haykowsky M., Scott J., Esch B., Schopflocher D., Myers J., Paterson I., Warburton D., Jones L., Clark A.M. (2011). A meta-analysis of the effects of exercise training on left ventricular remodeling following myocardial infarction: Start early and go longer for greatest exercise benefits on remodeling. Trials.

[59] Kraigher-Krainer E., Shah A.M., Gupta D.K., Santos A., Claggett B., Pieske B., Zile M.R., Voors A.A., Lefkowitz M.P., Packer M. (2014). Impaired systolic function by strain imaging in heart failure with preserved ejection fraction. J. Am. Coll. Cardiol..

[60] Buggey J., Alenezi F., Yoon H.J., Phelan M., DeVore A.D., Khouri M.G., Schulte P.J., Velazquez E.J. (2017). Left ventricular global longitudinal strain in patients with heart failure with preserved ejection fraction: Outcomes following an acute heart failure hospitalization. ESC Heart Fail..

[61] Park J.J., Choi H.M., Hwang I.C., Park J.B., Park J.H., Cho G.Y. (2019). Myocardial Strain for Identification of beta-Blocker Responders in Heart Failure with Preserved Ejection Fraction. J. Am. Soc. Echocardiogr..

[62] Park J.J., Mebazaa A., Hwang I.C., Park J.B., Park J.H., Cho G.Y. (2020). Phenotyping Heart Failure According to the Longitudinal Ejection Fraction Change: Myocardial Strain, Predictors, and Outcomes. J. Am. Heart Assoc..

[63] Kalogeropoulos A.P., Georgiopoulou V.V., Gheorghiade M., Butler J. (2012). Echocardiographic evaluation of left ventricular structure and function: New modalities and potential applications in clinical trials. J. Card. Fail..

[64] Collier P., Phelan D., Klein A. (2017). A Test in Context: Myocardial Strain Measured by Speckle-Tracking Echocardiography. J. Am. Coll. Cardiol..

[65] Malm S., Frigstad S., Sagberg E., Larsson H., Skjaerpe T. (2004). Accurate and reproducible measurement of left ventricular volume and ejection fraction by contrast echocardiography: A comparison with magnetic resonance imaging. J. Am. Coll. Cardiol..

[66] Segar M.W., Patel K.V., Ayers C., Basit M., Tang W.H.W., Willett D., Berry J., Grodin J.L., Pandey A. (2020). Phenomapping of patients with heart failure with preserved ejection fraction using machine learning-based unsupervised cluster analysis. Eur. J. Heart Fail..

